# Efficacy of endoscopic interlaminar decompression in lumbar spinal stenosis: a retrospective study

**DOI:** 10.1038/s41598-024-77337-2

**Published:** 2024-11-06

**Authors:** Xiao Qu, Lin Zhang, Zhou xie, Jun Zhang, Yanran Huang, Ningdao Li, Xiaoji Luo

**Affiliations:** 1 Department of Orthopedics, The First Affiliated Hospital of Chongqing Medical University, Chongqing, 400016 China; 2 Hainan Hospital of Traditional Chinese Medicine, Hainan, 570203 China; 3 Department of Orthopedics, The First Affiliated Hospital of Chongqing Medical and Pharmaceutical College, Chongqing, 400060 China

**Keywords:** Lumbar spinal stenosis, Percutaneous spinal endoscopic surgery, Interlaminar approach, Diseases, Medical research

## Abstract

This retrospective study evaluated the clinical effectiveness of endoscopic interlaminar decompression for lumbar spinal stenosis in 40 patients treated from February 2020 to January 2022. The procedure was successful in all cases, with only one dural sac injury reported and no other complications. Postoperative Visual Analogue Scale (VAS) and Japanese Orthopaedic Association (JOA) scores improved significantly (P<0.05), and the modified Macnab criteria showed an 87.5% rate of excellent and good outcomes at follow-up. No patients required revision surgery. Overall, the endoscopic interlaminar approach proved to be effective, safe, and supported early recovery.

## Introduction

In the face of an increasingly aging population, degenerative lumbar spine disease has emerged as a significant factor impacting the quality of life among the elderly. Specifically, degenerative lumbar spinal stenosis has emerged as a leading cause for spinal surgery in older individuals. This condition is typified by neurogenic claudication, lower back and leg pain, attributed to pathological processes such as hyperplasia and hypertrophy of the ligamentum flavum, facet joint capsule cohesion, facet hypertrophy, and intervertebral disc degeneration^1–3^When conservative treatments fail to alleviate symptoms, surgery becomes necessary.The primary objectives of surgery are to alleviate pain, mitigate functional impairment, and enhance quality of life. Current research in the field is primarily focused on minimizing muscle and soft tissue damage, reducing blood loss, and expedite postoperative recovery, while still achieving the same decompression objective, in comparison to conventional surgery.Percutaneous endoscopic interlaminar decompression (PEID) has demonstrated beneficial therapeutic outcomes in the treatment of conditions such as lumbar disc herniation^4,5^. Therefore, this study investigates the efficacy of a spinal endoscopic technique to treat lumbar spinal stenosis through an interlaminar approach, resulting in satisfactory clinical outcomes. Further details of the report follow.

## Materials and methods

### General Information

This study included a comprehensive review of 40 patients diagnosed with lumbar spinal stenosis, all of whom underwent spinal endoscopic surgery in our orthopedic department between February 2020 and January 2022. Among these patients, thirty-eight presented with single-segment stenosis, while two had double-segment stenosis, resulting in a total of 42 operative segments.Inclusion criteria were as follows: (1) Clear diagnosis of lumbar spinal stenosis, with primary symptoms being neurogenic claudication and lower limb pain; (2) Indication for surgical intervention through Percutaneous Endoscopic Interlaminar Decompression (PEID) following unsuccessful conservative treatment for a duration exceeding 3 months; (3) Post-operative follow-up extending up to 6 months, with a complete follow-up history available.Exclusion criteria included: (1) Presence of other spinal diseases, such as lumbar instability, spondylolisthesis, or isolated disc protrusion; (2) Previous history of spinal surgery or spinal revision surgery.This study was approved by the Institutional Review Board of The First Affiliated Hospital of Chongqing Medical University, and informed consent was obtained from all participating patients.all research was performed in accordance with relevant guidelines/regulations. Research involving human research participants have been performed in accordance with the Declaration of Helsinki.

### Surgical technique

**Fig. 1:**
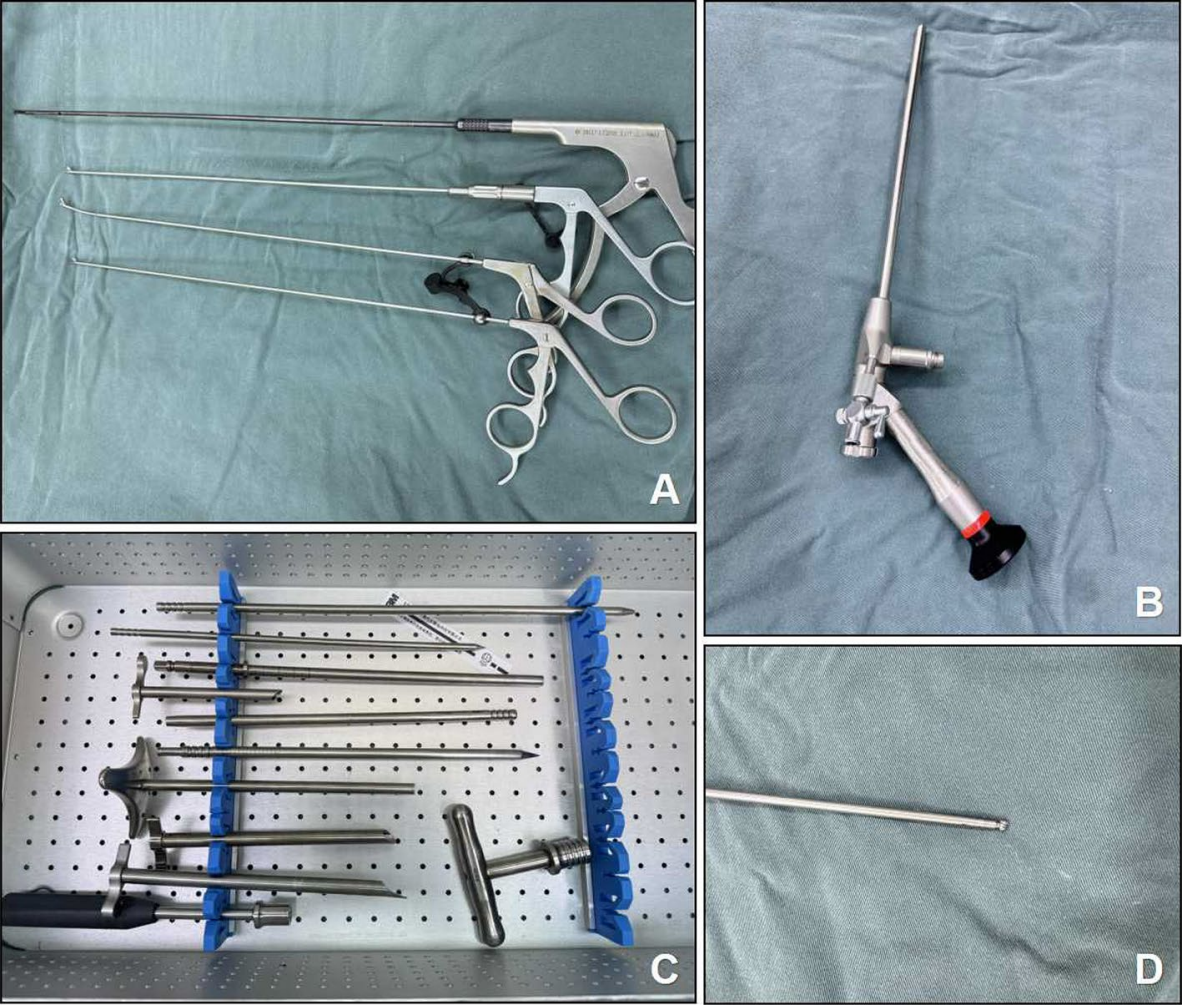
(**A**) Kerrison Rongeur 3.5 mm, Nucleus pulposus forceps 3 mm (**B**) Endoscope system for spinal stenosis(working channel outer diameter 6.3 mm, Inner working channel diameter is 3.75 mm) (**C**) Tapered dilator (**D**) Diamond drill 3.5 mm.

Patients were meticulously positioned prone with flexed knees and hips on a radiolucent surgical table. A G-type X-ray machine was used to identify the surgical site, followed by an application of a surgical marking approximately 1.5 cm lateral to the spinous process at the lower edge of the upper vertebral lamina of the operative segment. After disinfection and draping, 1% lidocaine was administered as a local anesthetic. A long needle, accompanied by a guide wire, was punctured down to the involved spinal level. The skin and fascia were incised with a no.10 scalpel. A tapered dilator was inserted along the guide wire to the bone surface, and a working cannula was introduced over the dilator to establish a portal. The endoscope was then inserted through the tube to carry out the ensuing procedures.Upon confirming correct endoscope placement under fluoroscopic guidance, a ring saw was used to grind at the surgical mark, representing the ligamentum flavum’s attachment point. The ring saw and drill were then used to detach the ligamentum flavum from this attachment point, comprising the entire interlaminar space. The ligamentum flavum was fully removed using a nucleus forceps.The lateral recess was examined and a drill was employed to enlarge this recess until sufficient decompression of the dura and nerve root was achieved. The ring saw and drill were applied to the facet on the same side, creating a space for the working cannula to rotate downwards to the posterior edge of the disc, enabling the removal of the herniated disc. Following the decompression of the dura and nerve root, the procedure was concluded. The working cannula was withdrawn, and the local wound was sutured with a stitch before a sterile dressing was applied. The surgical instruments used in this procedure (Fig. 1).

### Postoperative management

Postoperatively, regular dressing changes are conducted, with analgesic, anti-inflammatory, and neurotrophic medications being administered as necessary. No antibiotics were used.For a period of three months post-operation, patients were mandated to wear a waist brace and advised to stay mostly bed-bound for three weeks. Ambulation, not exceeding 20 min per session, was permitted as appropriate.From the end of the third week through to three months postoperatively, physical activity could be gradually escalated as long as it did not induce discomfort. Patients are advised to avoid bending and any other strenuous activities during this recovery period.

### Observation indicators and efficacy evaluation

Key parameters recorded included the duration of the endoscopic operation for each surgical segment and the length of the postoperative hospital stay. For comparative evaluation of clinical outcomes, the Visual Analogue Score (VAS) and the Japanese Orthopaedic Association Scores (JOA) were employed preoperatively and at the six-month postoperative mark. Furthermore, the MacNab score was used to evaluate clinical efficacy at the final follow-up conducted at the end of the six-month period.

### Statistical analysis

All statistical analyses were performed utilizing SPSS 26.0 software. For descriptive statistical analysis, count data was expressed as the number of cases and rates. Given that the measurement data adhered to the normal distribution, the independent sample t-test was employed for difference analysis. *P* < 0.05 was considered statistically significant.

## Results

The study included a total of 40 patients, comprising 29 males and 11 females, with ages ranging from 47 to 85 years. The mean age was calculated to be 63.45 ± 8.76 years. Among these patients, thirty-eight presented with single-segment disease, while two had double-segment disease. Specifically, the affected interspaces among patients with single-segment disease were as follows: L3-4 in 6 cases, L4-5 in 28 cases, and L5-S1 in 4 cases. Both cases of double-segment disease were reported at the L3-4 and L4-5 spaces, respectively. The preoperative Visual Analogue Score (VAS) and the Japanese Orthopaedic Association Scores (JOA) averaged 6.80 ± 0.79 and 11.83 ± 1.78, respectively.

All patients successfully underwent the operation. The duration of the Percutaneous Endoscopic Interlaminar Decompression (PEID) procedure ranged from 69 to 116 min, with an average duration of 83.11 ± 9.70 min. The postoperative hospital stay varied between 2 and 14 days, with an average stay of 3.18 ± 1.96 days. There was one instance of a dural sac injury, which was managed with deep suturing and pressure bandaging, and consequently recovered postoperatively. Every patient in this group had a minimum follow-up period of six months, and there were no instances of surgical revision during this period. A significant decrease in pre- and postoperative VAS scores was noted (*p* < 0.001), along with a significant increase in JOA scores (, *p* < 0.001).The modified MacNab score was utilized to evaluate the clinical outcome at the final follow-up: 17 cases were rated as excellent, 18 as good, and 5 as fair, with an overall success rate (excellent and good) of 87.5%.The sociodemographic data and follow-up information are delineated in Tables 1 and 2. A representative case is displayed in Fig. 1.

**Table 1:** Postoperative demographics and clinical characteristics.

Characteristics	Value
Gender	
Male Female	11 29
Age (year, x ± s)	63.45 ± 8.76
Surgical Spinal Segment(i.e., L34/L45/L45L34/L5S1)	8/28/2/4
Surgery duration (min, x ± s)	83.11 ± 9.70
Post-operative Hospital Stay (day, x ± s)	3.18 ± 1.96
MacNab score: 6 months post-operation (i.e., excellent/good/fair)	29/5/6

**Table 2:** Comparison of pre and post-operation (x ± s).

Timeline	VAS score	JOA score
Pre-operation	6.80 ± 0.79	11.83 ± 1.78
Post-operation	1.63 ± 0.67	25.70 ± 1.80
P-value	*p* < 0.001	*p* < 0.001

## Discussion

Due to the lack of high-level evidence, the treatment of degenerative lumbar spinal stenosis in elderly patients remains a subject of debate. Currently, the primary treatment options for this condition include conservative management and surgical intervention. For patients with mild symptoms, conservative treatments such as medication, physical therapy, and exercise therapy are recommended^6^. Among these, exercise therapy is currently recommended as a first-line conservative treatment for lumbar spinal stenosis^7^. Exercise therapy primarily includes lumbar flexion and extension exercises, core stability training, cycling exercises, body-weight-supported treadmill training (BWSTT), aquatic exercises, and other therapeutic exercises. For patients with lumbar spinal stenosis (LSS) who fail to respond to conservative treatment, surgery becomes the only option. However, traditional laminectomy still has several drawbacks, including the stripping of musculoligamentous attachments of the posterior spinal elements at the affected segment. This may lead to segmental instability, muscle weakness, and postoperative pain^8–10^. Although spinal surgery has gradually moved towards minimally invasive techniques, such as MIS TLIF and OLIF, these procedures still pose challenges, including significant bodily trauma, prolonged bed rest, and reduced quality of life^11,12^. Since the introduction of spinal endoscopic surgery in China in the late 1990s, it has gained popularity among surgeons due to its ability to minimize muscle and soft tissue damage, thereby reducing intraoperative bleeding, alleviating postoperative pain, shortening postoperative bed rest, and decreasing average hospital stay. As this technology has continued to evolve, various innovative surgical techniques have emerged. While this is beneficial for patients, it also poses challenges for surgeons in making clinical decisions.

Reports have indicated that the transforaminal approach for treating lumbar spinal stenosis has achieved commendable results. However, minimally invasive spinal surgery using full endoscopic techniques presents certain challenges when addressing conditions other than herniated discs, such as spinal stenosis^13^. Among the various techniques for treating lumbar spinal stenosis, the interlaminar approach is considered a reasonable choice for central stenosis, as opposed to the transforaminal approach. Research has shown that in patients with primary neurogenic claudication without instability or a history of previous surgery, adequate decompression using full endoscopic techniques via the interlaminar approach can yield good clinical outcomes. However, due to factors such as limited surgical visualization and the technical skills required, achieving complete decompression of the lateral recess and contralateral dorsal nerve roots remains challenging, which continues to limit the widespread application of this technique^14^. In our surgical procedures, the development of minimally invasive instruments, such as powered drills and visualized trephines, has facilitated the decompression of posterior ligamentous structures and hypertrophic facets, making bilateral decompression via a unilateral approach feasible. In this study, decompression was performed along the insertion and origin of the ligamentum flavum between the laminae, simplifying the surgeon’s task in defining the range of decompression and delineating the surgical path. This method, through direct visualization, reduces the risks of incomplete decompression, dural sac injury, and nerve root injury due to unclear anatomical relationships. We utilized a new endoscopic system with an outer diameter of 6.3 mm and a working channel diameter of 3.75 mm, which allows for adequate bilateral decompression while significantly minimizing patient trauma. This surgery was performed under local anesthesia with the sole use of intravenous dexmedetomidine infusion, which is safer for patients compared to general anesthesia. Patients were able to engage in light activities as early as three hours postoperatively, and no postoperative drainage tubes were required. The procedure significantly shortened the operation time and minimized trauma, leading to a quicker recovery.Surgical interventions are inevitably come with potential complications. As per existing literature, the most prevalent complications include persistent postoperative pain, rupture of the dural sac, the necessity for spinal revision surgery due to recurrence, and instability induced by excessive resection of facet joints^15,16^. In our cohort, one case of dural sac injury was recorded, likely due to the dura mater adhering to the ligamentum flavum. To prevent adverse outcomes, deep suturing and pressure bandaging were employed postoperatively.Overall, our study highlights the efficacy and safety of endoscopic interlaminar decompression as a minimally invasive therapeutic option for lumbar spinal stenosis, particularly in elderly patients. While surgical interventions carry inherent risks, advancements in surgical techniques and careful attention to surgical details can help mitigate these risks and improve patient outcomes. In conclusion, our study highlights the efficacy and safety of endoscopic interlaminar decompression as a minimally invasive treatment option, particularly in elderly patients.

## Conclusions

In conclusion, percutaneous endoscopic interlaminar decompression emerges as secure, effective, and minimally invasive therapeutic avenue for lumbar spinal stenosis. Its advantages, including minimal, incision, reduced bleeding, feasibility under local anesthesia, and swift recoveryAlthough its short-term efficacy appears promising, continued observation is essential to ascertain its long-term outcomes. Further research and follow-up studies are warranted to comprehensively evaluate its lasting benefits and potential complications. Nonetheless, its current benefits position it as a valuable alternative in the treatment armamentarium for lumbar spinal stenosis, offering patients a minimally invasive yet effective therapeutic option.

**Fig. 2:**
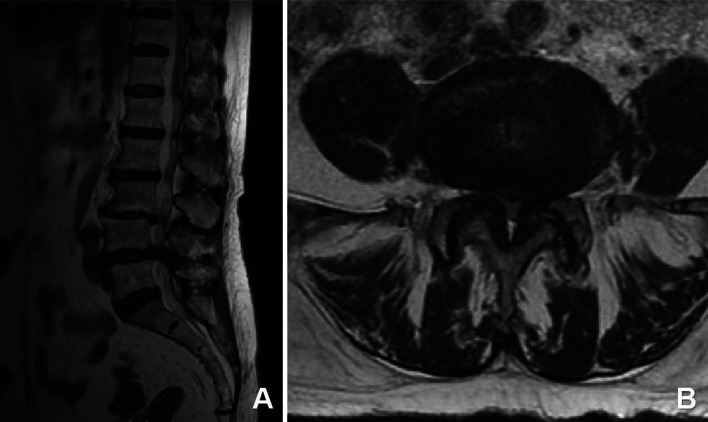
Preoperative magnetic resonance imaging (MRI) examination revealed the presence of spinal stenosis at the L4/5 level.

**Fig. 3:**
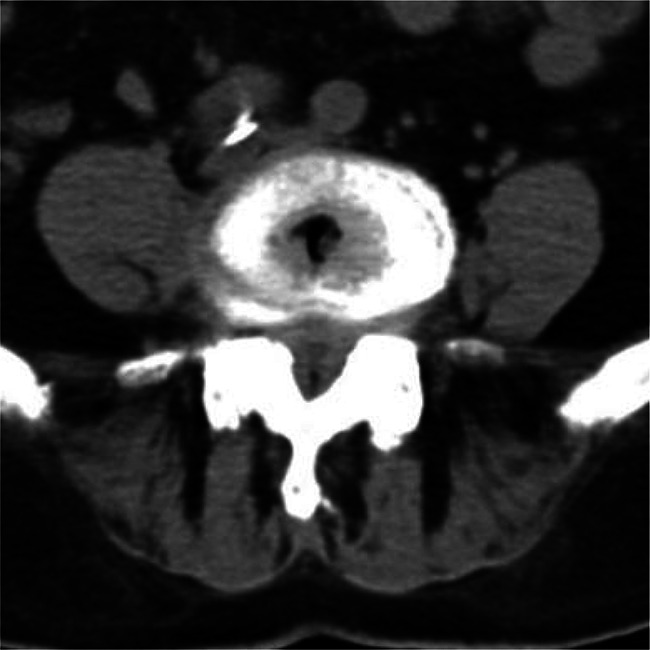
Preoperative computed tomography (CT) examination confirmed the diagnosis of spinal stenosis.

**Fig. 4:**
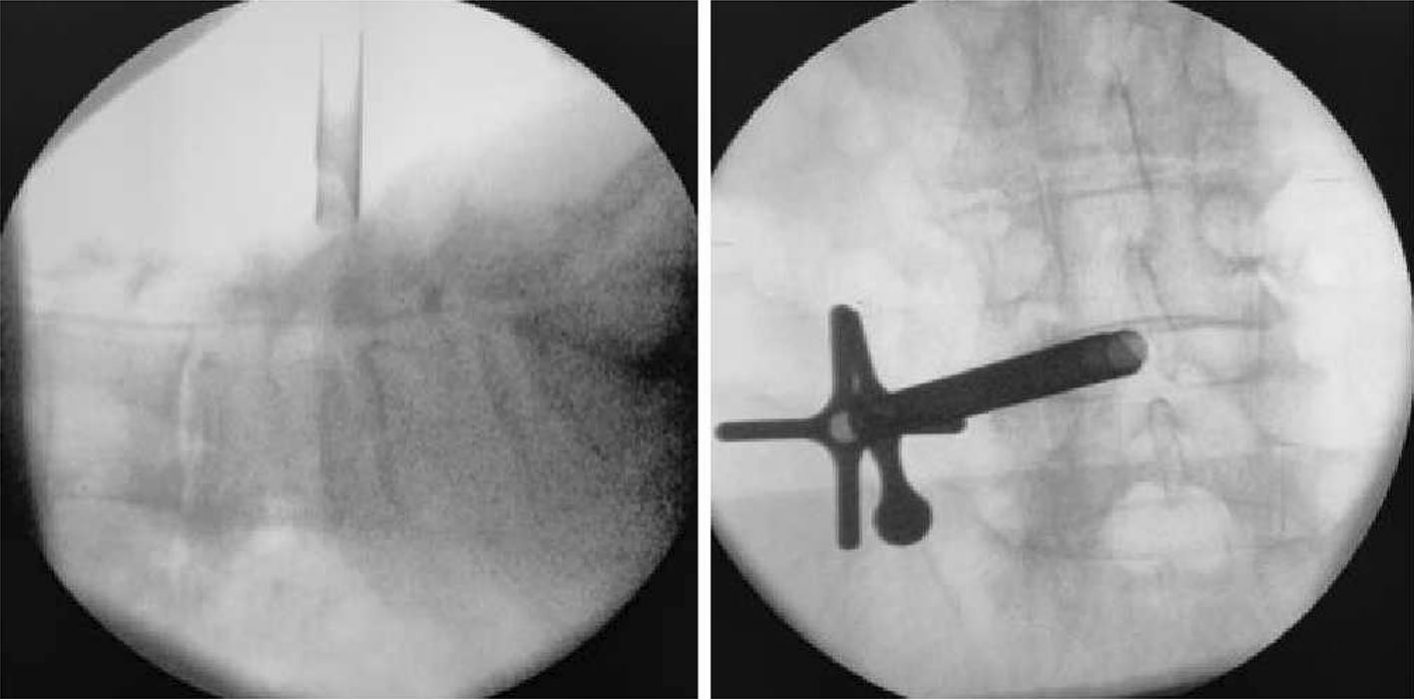
Intraoperative fluoroscopy images depict the precise placement of the ring saw at the designated location, guided by endoscopic visualization.

**Fig. 5:**
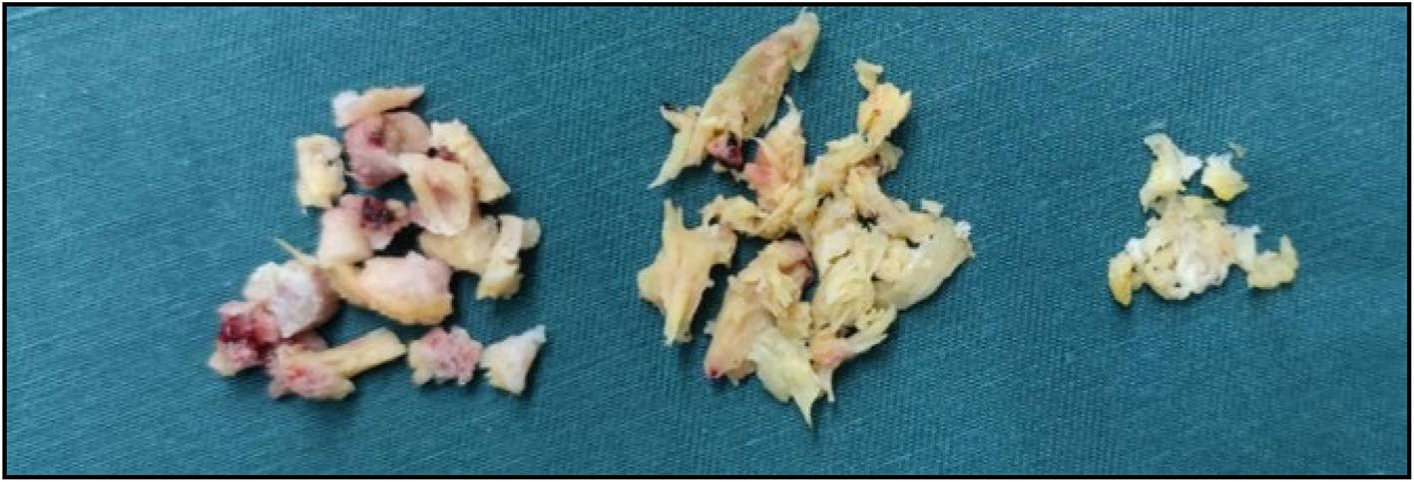
A surgical specimen was obtained, displaying the excised bone, ligamentum flavum, and intervertebral disc tissue.

**Fig. 6:**
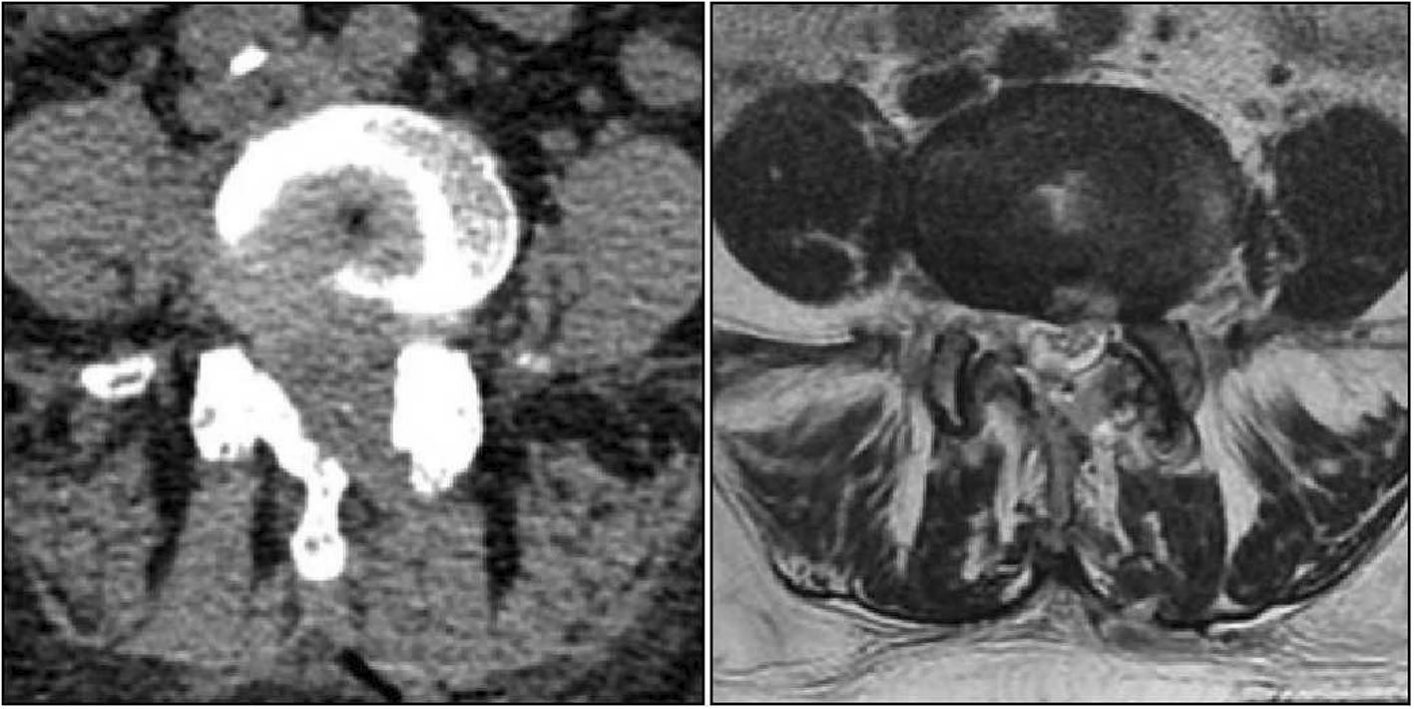
Postoperative follow-up MRI and CT scan demonstrated a significant increase in spinal canal volume when compared to the preoperative imaging.

Patient: Female, 82 years old.Chief Complaint: The patient presented with a history of neurogenic claudication for over 10 years, which had worsened over the past 3 months with bilateral leg pain. Subsequently, the patient underwent spinal endoscopic surgery through the percutaneous endoscopic interlaminar decompression (Figs. 2, 3, 4, 5 and 6).

## Data Availability

The datasets used and/or analyzed during the current study are available from the corresponding author on reasonable request.
